# Optimizing Acute Coronary Syndrome Patient Treatment: Leveraging Gated Transformer Models for Precise Risk Prediction and Management

**DOI:** 10.3390/bioengineering11060551

**Published:** 2024-05-29

**Authors:** Yingxue Mei, Zicai Jin, Weiguo Ma, Yingjun Ma, Ning Deng, Zhiyuan Fan, Shujun Wei

**Affiliations:** 1 People’s Hospital of Ningxia Hui Autonomous Region, Ningxia Medical University, Yinchuan 750101, China; myxgk2@163.com (Y.M.); maweiguo_87@126.com (W.M.); m13995050810@outlook.com (Y.M.); 2 Tongxin County People’s Hospital, Wuzhong 751309, China; txwkjzc@126.com; 3 College of Biomedical Engineering and Instrument Science, Ministry of Education Key Laboratory of Biomedical Engineering, Zhejiang University, Hangzhou 310027, China; zju.dengning@gmail.com; 4 Centre of Intelligent Medical Technology and Equipment, Binjiang Institute of Zhejiang University, Hangzhou 310053, China; frank.zhiy@gmail.com

**Keywords:** acute coronary syndrome, major adverse cardiovascular events, transformer, machine learning, risk assessment, patient management

## Abstract

Background: Acute coronary syndrome (ACS) is a severe cardiovascular disease with globally rising incidence and mortality rates. Traditional risk assessment tools are widely used but are limited due to the complexity of the data. Methods: This study introduces a gated Transformer model utilizing machine learning to analyze electronic health records (EHRs) for an enhanced prediction of major adverse cardiovascular events (MACEs) in ACS patients. The model’s efficacy was evaluated using metrics such as area under the curve (AUC), precision–recall (PR), and F1-scores. Additionally, a patient management platform was developed to facilitate personalized treatment strategies. Results: Incorporating a gating mechanism substantially improved the Transformer model’s performance, especially in identifying true-positive cases. The TabTransformer+Gate model demonstrated an AUC of 0.836, a 14% increase in average precision (AP), and a 6.2% enhancement in accuracy, significantly outperforming other deep learning approaches. The patient management platform enabled healthcare professionals to effectively assess patient risks and tailor treatments, improving patient outcomes and quality of life. Conclusion: The integration of a gating mechanism within the Transformer model markedly increases the accuracy of MACE risk predictions in ACS patients, optimizes personalized treatment, and presents a novel approach for advancing clinical practice and research.

## 1. Introduction

Acute coronary syndrome (ACS) is identified as the most severe category within coronary heart diseases, characterized by a range of symptoms resulting from diminished coronary artery blood flow. This impairment can lead to the malfunction or death of heart muscle tissue [1]. The “2022 Heart Disease and Stroke Statistical Report” by the American Heart Association, drawing on 2019 data, revealed a mortality rate of 214.6 per 100,000 individuals in the United States due to cardiovascular diseases. Statistically, cardiovascular disease causes a death every 36.1 s, totaling 2396 deaths daily. Notably, for individuals aged 45 and above, the mortality rate one year post-first myocardial infarction is reported at 18% for males and 23% for females [2]. Concurrently, in China, there is a year-over-year increase in ACS incidence, with the patient count exceeding 2.5 million, thereby becoming the foremost cause of mortality among both urban and rural populations [3]. The “China Cardiovascular Health and Disease Report 2022” indicates that in 2020, cardiovascular diseases accounted for 48.00% and 45.86% of all deaths in rural and urban areas, respectively, highlighting them as the primary mortality cause. Annually, China records nearly one million new ACS cases. The abrupt onset, grave severity, and intricate progression of ACS frequently culminate in death or disability due to acute myocardial ischemia [4], thereby imposing a significant challenge on China’s public health and healthcare systems.

In the early management of ACS, accurately determining patients’ risk levels for MACEs, such as myocardial infarctions, strokes, or death, is crucial. This phase of risk assessment enables physicians to tailor treatment strategies effectively, thereby reducing ACS mortality rates. Widely adopted in clinical settings are the Thrombolytic Therapy for Myocardial Infarction (TIMI) [5,6], the Global Registry of Acute Coronary Events (GRACE) [7,8], and the CRUSADE* bleeding risk assessment [9] tools. These traditional scoring mechanisms, generally derived from longitudinal cohort studies, offer substantial reliability. They track participants over extended periods to collect objective data on adverse outcomes, providing a vital evidence base for understanding cardiovascular disease etiology and evaluating diagnostic and therapeutic interventions. However, their effectiveness is constrained by a limited array of considered risk factors and the stringent inclusion criteria of cohort studies, which may not fully represent the broader clinical context. Additionally, the lengthy duration required to conclude these studies introduces challenges in maintaining consistent patient data collection. Moreover, despite the extensive use of these risk assessment tools in practice, their limitations in patient management are increasingly apparent. Particularly notable is the gap in integrating a digital patient management system, which leads to data fragmentation, inadequate real-time monitoring, and obstacles in personalizing treatment regimens [10]. Traditional risk evaluation tools depend on static data, neglecting the potential for patients’ conditions to evolve [11]. This static methodology hampers the ability of healthcare providers to accurately gauge current risk levels, potentially delaying or compromising treatment decisions. Furthermore, the lack of digital support complicates the personalization of treatment, a necessity in the era of precision medicine, which demands tailoring strategies to individual patient profiles, including lifestyles and medical history [12]. Without access to comprehensive, actionable patient data, developing and implementing personalized treatment plans faces considerable hurdles.

In recent years, the advancement of hospital informatization has paved the way for the development of sophisticated machine learning models leveraging electronic health record (EHR) data. These models are designed to mirror the complexity of the clinical environment closely and integrate a broader spectrum of risk factors. Wu et al. [13] analyzed data from 536 cases at the Geisinger Clinical Center to predict heart failure occurrence within six months prior to a formal diagnosis, employing various methodologies, including logistic regression, support vector machines, and boosting, complemented by diverse feature selection techniques. Similarly, Syed et al. [14] introduced a similarity-based unsupervised algorithm for the risk stratification of patients facing major adverse cardiac events, demonstrating its efficacy in identifying individuals at double the risk of such events within a 90-day timeframe. Further, Churpek et al. [15] developed a discrete-time multinomial logistic regression model targeting cardiac arrest events, which, by utilizing vital signs, demographic data, and test results from EHRs, outperformed traditional models relying solely on vital signs in predictive accuracy. Dong et al. [16] innovated by integrating genetic algorithms with fuzzy association rules to create an intelligent system for assessing the risk of unstable angina, wherein fuzzy sets are utilized to model the imprecision of input data, facilitating the extraction of a set of fuzzy rules for a risk evaluation. Verma [17] proposed a novel hybrid algorithm for a computer-aided diagnosis that combines effective feature selection, particle swarm optimization search techniques, and K-means clustering algorithms for risk factor identification. Krittanawong et al. [18] explored the utilization of artificial intelligence in identifying cardiovascular disease risk factors, optimizing treatment approaches, and forecasting patient outcomes, suggesting that a holistic digital management and data analysis approach could offer patients more customized treatment plans, thus enhancing therapeutic outcomes and patient satisfaction. Muthukumarasamy et al. developed a cloud-based tool to enhance ACS management by focusing on the revascularization of non-culprit lesions. Popa-Fotea et al. [19] demonstrated that machine learning improves diagnostic accuracy and treatment effectiveness across diverse diseases [20]. Lastly, Johnson et al. [21] focused on the extensive application of artificial intelligence in cardiology, encompassing the automatic detection of arrhythmias, the risk assessment of myocardial infarction, and the early diagnosis of cardiovascular diseases, noting that the integration of multiple machine learning techniques, such as logistic regression, support vector machines, and deep learning, can significantly augment the accuracy of cardiovascular disease predictions and diagnoses.

In summary, unlike traditional models based on cohort studies, data-driven research methods using structured EHR can handle a large number of cases without strict inclusion or exclusion criteria, reflecting the real clinical environment and more conveniently incorporating new risk factors. However, predicting MACEs in ACS is a typical imbalanced learning problem. Identifying outcomes from minority class samples correctly and considering potential associations among samples and features are challenges that need to be addressed. This paper proposes a Transformer model based on a gating mechanism aimed at improving the prediction of MACEs in patients with ACS. First, we utilized a TabTransformer [22] based on the Transformer architecture to process structured tabular data, effectively capturing potential relationships between samples and understanding and predicting interactions among complex biomarkers related to the disease. We then introduced a gating mechanism to solve the imbalanced learning problem. The gating mechanism regulates the flow of information, enhancing the model’s learning from minority class features, thus improving the identification of minority class samples without sacrificing the overall performance. Finally, a management platform for ACS patients was established around the TabTransformer model with the gating mechanism. This platform is used for risk prediction of major cardiovascular adverse events and recommends personalized management and treatment strategies based on the specific conditions of the patients. Through this approach, we aim to improve the treatment outcomes for ACS patients, reduce the incidence of major adverse cardiovascular events, and ultimately enhance the quality of life for patients.

## 2. Materials and Methods

### 2.1. Data Preprocessing

Data Collection. This study analyzed a comprehensive dataset containing records of cardiovascular inpatients collected from January 2014 to December 2019 at a hospital in Ningxia. The dataset includes six categories of real data: basic information, medical history, laboratory test results, follow-up data, echocardiogram (ECHO) data, and angiography data. The dataset contains structured basic information, medical history, laboratory test results, echocardiogram data, and angiography data, while follow-up data only retained the data structure without data instances.

Data Processing and Analysis. The data underwent rigorous preprocessing to ensure consistency, accuracy, and integrity of the analysis. The following preprocessing methods were applied:

Numerical Data: Numerical data were standardized by subtracting the mean and dividing by the standard deviation [23], and data were normalized between 0 and 1 to mitigate biases caused by different scales and distributions.

Categorical Data: Categorical variables were converted into binary columns, with each category represented by an independent column, using 1 to indicate presence and 0 to indicate absence [24]. A unique integer was assigned to each category.

Time Series Data: Features such as year, month, day, day of the week, and holiday flags were extracted to capture cyclical changes over time [25]. Sliding windows were used to generate historical aggregates to capture dynamic changes in time series data.

Handling Missing Data: Missing values were filled using the mean, median, mode, or model predictions [26]. Rows or columns with missing values were deleted when there were minimal missing data.

Outlier Handling: Data points significantly different from others were excluded [27].

These preprocessing steps ensured that the neural network model would not be biased due to differences in scale, missing values, or outliers.

### 2.2. Table Data Modeling Based on Gated Controllers

**Figure 1:**
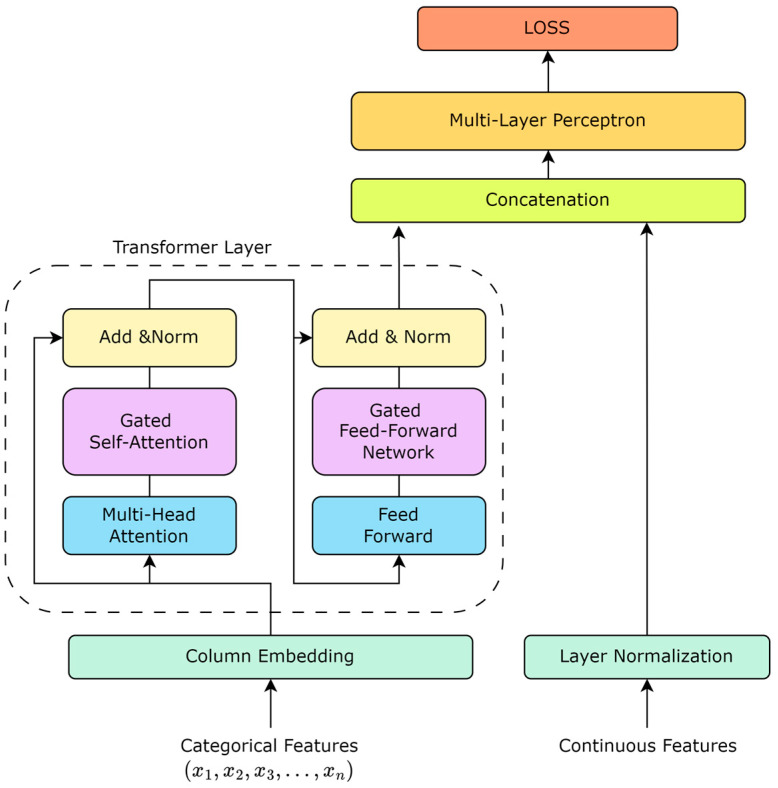
TabTransformer structure based on gate mechanism.

The TabTransformer rethinks the application of the Transformer architecture to tabular data, offering an effective way to encode categorical features into meaningful representations. Unlike traditional processing methods, where categorical features are treated as independent entities, the TabTransformer introduces the concept of context embeddings, allowing for the model to capture dependencies between features, thereby deepening its understanding of the data. Based on this, as shown in Figure 1, our work enhances the TabTransformer architecture by incorporating a gating mechanism into the self-attention mechanism. The introduction of the gating mechanism aims to improve the model’s dynamic adjustment capability for feature importance, enabling the model to identify and utilize the interactions between key features more flexibly.

Algorithm 1 outlines the steps for the gated Transformer model designed for MACE risk prediction. The procedure begins with the initialization of the dataset, which contains EHR data from ACS patients, followed by preprocessing to extract relevant features. The model, featuring a series of Transformer layers augmented with a gating mechanism, processes each feature set. This gating mechanism dynamically adjusts the attention outputs by applying calculated gating coefficients, which allow for precise modulation of the information flow based on the features’ significance. The model undergoes iterative training until convergence, ensuring adaptability and robust generalization across varied datasets. This method enhances the model’s accuracy in predicting MACE risk, making it highly applicable for clinical settings.
**Algorithm 1:** Gated Transformer Model for MACE Risk Prediction1:   *Initialize* dataset **D** containing EHR data of ACS patients2:   *Preprocess*
**D** to extract features **F** for each patient3:   *Initialize* the gated Transformer model **M**4:   Define gate structure within **M** that adjusts information flow5:   **while** model **M** has not converged **do**6:   **for** each batch **B** from **F do**7:     **for** each feature set **f** in **B do**8:       Encode **f** using input embeddings to receive **E**9:       **for** each Transformer layer **L** in **M do**10:         Apply self-attention to **E** to receive attention outputs **A**11:         Apply gate **G** to **A**:12:           $\mathrm{Compute}\;\mathrm{gating}\;\mathrm{coefficients}\;G_{c}=$
$sigmoid(W_{g}*A+b_{g})$
13:            
$\mathrm{Adjust}\;A\;\mathrm{using}\;G_{c}$
$\;\mathrm{to}\;\mathrm{receive}\;\mathrm{gated}\;\mathrm{outputs}\;G_{A}=G_{c}⨀A$
14:         $\mathrm{Pass}\;G_{A}$ to the next layer or output layer15:       **end for**16:     **end for**17:     Calculate predictions **P** for **B** using final outputs from **M**18:     Compute loss **L** between **P** and actual outcomes of **B**19:     Update weights of **M**, including gate parameters, to minimize **L**20:   **end for**21:   Validate **M** on a separate validation set and adjust if necessary22:   **end while**23:   $\mathrm{Deploy}\;M\;\mathrm{for}\;\mathrm{evaluation}\;\mathrm{on}\;a\;\mathrm{testing}\;\mathrm{set}\;F_{test}$
24:   $\mathrm{for}\;\mathrm{each}\;\mathrm{patient}\;p\;\mathrm{in}\;F_{test}$ **do**25:   $\mathrm{Predict}\;\mathrm{risk}\;R_{p}$ using **M** for **p**’s features26:   $\mathrm{Output}\;R_{p}$ indicating MACE risk27:   **end for**Ensure: High-accuracy MACE risk prediction incorporating dynamic gating

#### 2.2.1. Pre-Training Embedding

The pre-training of embeddings was carried out by pre-training the embedding layers on related tasks, allowing for the transfer of knowledge between different tasks and improving the model’s performance on the target task. A common method for pre-training is to use unsupervised learning, such as autoencoders, to learn rich embeddings that represent the features. Suppose there is an autoencoder whose encoder part maps the input feature $x_{i}$ to a hidden representation $h_{i}$, and then, the decoder part tries to reconstruct the original input, outputting ${\hat{x}}_{i}$. The operations of the encoder and decoder can be expressed by the following formulas:

(1) $$h_{i}=f\left(W_{e}x_{i}+b_{e}\right)$$

(2) $${\hat{x}}_{i}=g\left(W_{d}h_{i}+b_{d}\right)$$

Here, f and g are activation functions; $W_{e}$ and $W_{d}$ are the weight matrices for the encoder and decoder, respectively; and $b_{e}$ and $b_{d}$ are their biases.

The objective of pre-training is to minimize the difference between the input feature $x_{i}$ and the reconstructed feature ${\hat{x}}_{i}$, typically using a loss function, such as mean squared error (MSE):

(3) $$Loss=\frac{1}{N}\sum_{i=1}^{N}{\left\|x_{i}-{\hat{x}}_{i}\right\|}^{2}$$

After pre-training is completed, the weights $W_{e}$ and biases $b_{e}$ from the encoder part can be used to initialize the embedding matrix for the corresponding features in the TabTransformer. This method of pre-trained embeddings can help the model capture richer feature representations early in training, thereby improving learning efficiency and performance on the final task.

#### 2.2.2. Transformer Layer

The Transformer layer is the cornerstone of the TabTransformer architecture, facilitating the generation of contextual embeddings for tabular data. It consists of multiple self-attention mechanisms and position-wise feed-forward networks.

Self-Attention Mechanism: Each self-attention mechanism computes the attention scores between all pairs of positions in the input sequence, enabling the model to focus on different parts of the sequence for different tasks. The attention function is defined as follows:

(4) $$Attention\left(Q,K,V\right)=softmax\left(\frac{QK^{T}}{\sqrt{d_{k}}}\right)V$$

where Q, K, and V are the queries, keys, and values matrices, respectively, and $d_{k}$ is the dimensionality of the keys.

Position-wise Feed-Forward Networks: Following the self-attention mechanism, each position in the input sequence is processed by a feed-forward network, applied independently. This network consists of two linear transformations with a ReLU activation in between.

Residual Connection and Layer Normalization: Both the self-attention and feed-forward networks incorporate residual connections followed by layer normalization, enhancing the flow of gradients and stabilizing the training process.

#### 2.2.3. Column Embedding

For each categorical feature, an embedding layer maps each unique category to a high-dimensional space where the relationships between categories can be learned and represented more effectively. These embeddings serve as the input to the Transformer layers, facilitating the generation of context embeddings. For each categorical feature $c_{i}$, its embedding representation can be obtained by looking up the embedding matrix E, where each row of E represents the embedding vector of a category. If the index corresponding to the categorical feature $c_{i}$ is j, then its embedding vector $e_{i}$ can be obtained using the following formula: $e_{i}=E[j]$. For a feature with N possible categories, the embedding matrix E will be an N×D matrix, where D is the embedding dimension. In this way, each categorical feature is transformed into a D-dimensional vector, which is then fed into the Transformer layers of the TabTransformer.

#### 2.2.4. Gated Self-Attention Mechanism

For the output of the attention mechanism A, the gating mechanism is applied:

(5) $$G=\sigma \left(XW_{g}+b_{g}\right)$$

where $W_{g}\;$ is the gating weight matrix, $b_{g}\;$ is the bias term, and σ  is the Sigmoid activation function that produces gating coefficients G  ranging from 0 to 1, used to regulate the flow of information. The final gated self-attention output can be represented as: $A^{\prime }=G⨀A$, where ⨀  denotes element-wise multiplication. Through this method, the model can dynamically adjust the contribution of each feature.

#### 2.2.5. Gated Feed-Forward Network

In the standard feed-forward network (FFN), the gating mechanism should actually be integrated into the information flow to regulate and optimize the passage of information. Introducing a gating coefficient $G_{F}$ in the FFN aims to modulate the output after the nonlinear activation function, ensuring that only important signals can pass through:

(6) $$F=ReLU\left(A^{\prime }F_{1}+b_{1}\right)$$

where $A^{\prime }$ is the output from the previous layer (such as the gated self-attention layer) and $F_{1}$ and $b_{1}$ are the weights and biases of the first layer of the FFN.

Similar to the gated self-attention mechanism, the gated FFN also employs a gating mechanism to regulate the flow of information. The calculation of the gating coefficient is as follows:

(7) $$G_{F}=\sigma \left(A^{\prime }F_{g}+b_{gF}\right)$$

Here, $F_{g}\;$ and $b_{gF}$ are the weights and biases used for calculating the gating coefficient, and σ is the activation function (usually sigmoid) that ensures the output values are between 0 and 1, representing the degree to which each feature is allowed to pass.

After calculating the gating coefficient $G_{F}$, it is applied to the output of the FFN as follows:

(8) $$F^{\prime }=F⨀G_{F}$$

This allows for the model to dynamically adjust the amount of information passing through the feed-forward network, enhancing the model’s expressiveness and generalization ability.

### 2.3. Design of Acute Coronary Syndrome Patient Management Platform

**Figure 2:**
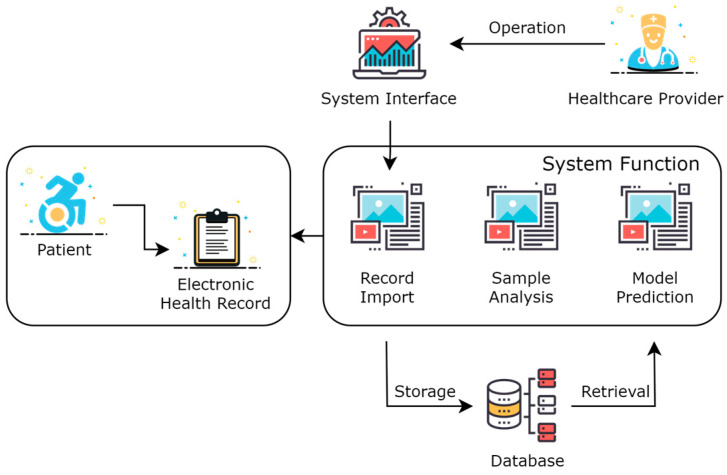
System architecture of acute coronary syndrome management platform.

As shown in Figure 2, this was the overall framework structure of the system. Healthcare providers operate visually through a system interface in the form of a Web website. The healthcare provider logs into the website, selects patients online, imports and visualizes the EHR of patients, and calls the model prediction service to perform adverse event predictions for the patient online. The data are stored in a database, which includes the imported EHR of patients, extracted feature data, model data, predicted result data, and intermediate process data.

EHR are collected with the patient as the basic unit, indexed by a unique patient ID, which can automatically associate the information of the patient’s past hospital stays. A patient corresponds to one or multiple hospital admissions. The diagnosis and treatment actions and the examination results of each admission are imported based on the hospitalization ID. After the electronic health record is imported into the system, it can be browsed and analyzed. Medical staff can use the models already loaded into memory to predict adverse events for patients. For example, to predict the occurrence of major cardiovascular adverse events, the system will automatically extract corresponding key factors from the entered patient information and make predictions. Particularly, medical personnel can also choose surgical or pharmaceutical interventions to predict the effect of intervention treatments on the patient.

The system’s web page was written based on Vue 3.3.0, the backend service was written in Java 1.8 based on Spring Boot, and the prediction model was set up using Python 3.9 based on the Flask framework.

## 3. Results

### 3.1. Results of Data Processing

After rigorous processing of 8583 data entries, we obtained 8005 training data entries. As shown in the table, the data are divided into six categories, comprising 134 variables: 8 items of basic information, 31 items of medical history, 30 items of laboratory test results, 25 items of follow-up data, 20 items of ECHO data, and 20 items of angiography data. The percentages shown in Table 1 represent the proportion of each data type relative to the total number of variables.

These proportions demonstrate the comprehensive nature of the dataset, ensuring that all relevant aspects of a patient’s medical profile are included. Each feature was verified by clinical experts to ensure accuracy and relevance to the study.

All six categories of data underwent preprocessing as described in Section 2.1. This included the normalization of numerical data, the binary encoding of categorical data, the handling of missing values, and outlier detection. This preprocessing ensured that the data were consistently formatted and free from biases that could affect model performance. The structured data were then presented to the model in a tabular format, capturing all relevant details in a standardized manner. This includes both binary classification variables and continuous numerical variables.

A complete set of the feature variables used in this study is provided in Appendix A. This appendix includes the names, types, and descriptions of each feature.

### 3.2. Comparison of Classification Results

In this study, the gold standard for comparison was a clinical assessment provided by a cardiologist. These experts reviewed patient data, including medical history, laboratory test results, and imaging data, from the 134 indicators in Table A1 to determine the risk of MACEs based on their clinical judgment and experience. Predictions made by different machine learning models are then compared to these expert assessments to assess their accuracy and effectiveness. This comparison ensures that the model’s predictions are consistent with established clinical practice and standards.

**Figure 3:**
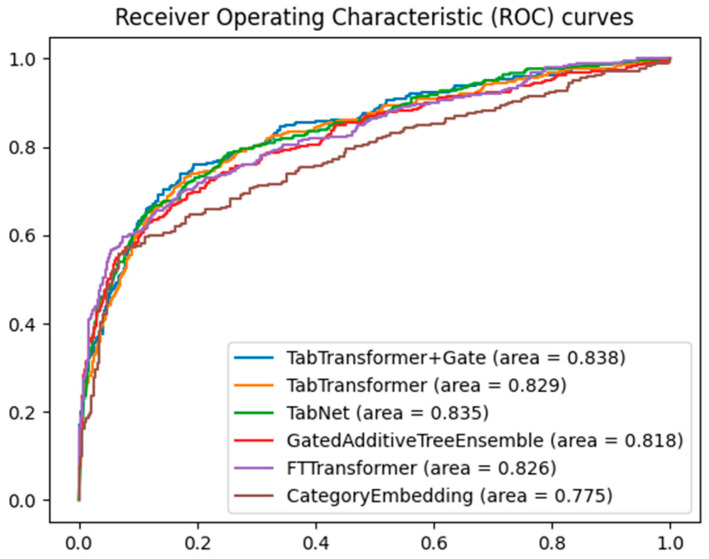
Comparative ROC curves for multiple models.

When evaluating the performance of different machine learning models on classification tasks, the receiver operating characteristic (ROC) curve and area under the curve (AUC) are widely used metrics. Figure 3 shows the ROC curves of five models for predicting major adverse cardiovascular events, namely the TabTransformer+Gate, TabTransformer, TabNet [28], GatedAdditiveTreeEnsemble [29], FTTransformer [30], and CategoryEmbedding [31] model, with the training parameters for the TabTranformer+Gate and TabTransformer presented in Table 2.

By comparing the area under the curve, it can be clearly seen that the TabTransformer+Gate model shows significant performance advantages. This result can be attributed to the model’s enhanced capability in feature selection and representation through the integration of the gating mechanism. The gated network can dynamically adjust the contribution of different features, optimizing feature representation, thereby achieving higher accuracy in classification tasks. Furthermore, the gating mechanism may also help the model achieve better generalization across different data subsets, reducing the risk of overfitting.

Second, the TabTransformer model without the gate mechanism and the FTTransformer model also exhibited high AUC values. Although there is a slight gap compared to the TabNet model, this demonstrates the Transformer architecture’s good representational learning ability in handling tabular data, effectively capturing long-distance dependencies between features through the self-attention mechanism. The TabNet model, with its sparse attention mechanism for feature selection, showed competitiveness on tabular datasets. Nonetheless, there is still room for improvement in terms of feature selection accuracy and model complexity.

The AUC value of the GatedAdditiveTreeEnsemble model, although lower than the aforementioned models, also showed relatively good performance. This indicates that hybrid methods combining ensemble tree models and deep learning models can improve classification effectiveness to some extent, even if they may not perform as well as purely deep learning methods. The CategoryEmbedding model had the lowest AUC value, which might indicate certain limitations of this model on the current dataset. This could be related to the model’s inability to fully capture complex patterns or feature interactions in the data, pointing out the need for improvement in its representation learning capability.

Gating mechanisms are particularly well-suited to Transformer architectures due to their ability to dynamically adjust the importance of each feature during training. The self-attention mechanism in Transformers dynamically weighs different parts of the input sequence, and the gating mechanism enhances this by controlling the flow of information, ensuring the model focuses on the most relevant features. Additionally, hierarchical processing through multiple Transformer layers benefits from the gating mechanism’s regulation of information flow, enhancing the model’s ability to learn hierarchical representations. This combination is crucial for tabular data with complex, multi-dimensional feature relationships.

**Figure 4:**
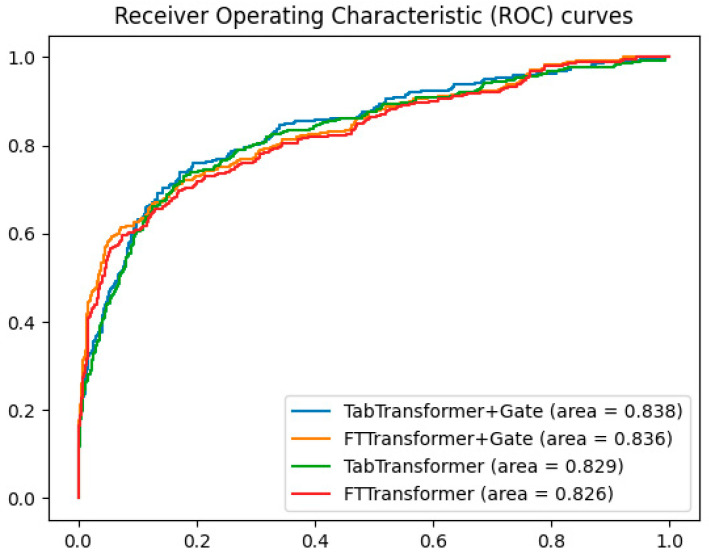
Comparison of gating mechanisms in transformer models.

To validate the effectiveness of the gating mechanism, additional experiments compared the ROC curves of the TabTransformer+Gate, TabTransformer, FTTransformer+Gate, and FTTransformer models. The results, shown in Figure 4, indicate that the TabTransformer+Gate model demonstrates superior performance with an AUC of 0.838. The FTTransformer+Gate model also shows improvement over the standard FTTransformer, achieving an AUC of 0.836 compared to 0.826. These findings suggest that while the gating mechanism benefits both models, it is particularly effective for the TabTransformer architecture.

In summary, the experimental results showcase the potential of using Transformer-based models on tabular data while highlighting the superior performance of the TabTransformer+Gate model in classification tasks on tabular data.

When evaluating the performance of classification models, the precision–recall (PR) curve is an important complement to the ROC curve, especially in cases of imbalanced data distribution. The PR curve illustrates the relationship between precision and recall at different thresholds, intuitively showing the model’s ability to classify minority classes.

**Figure 5:**
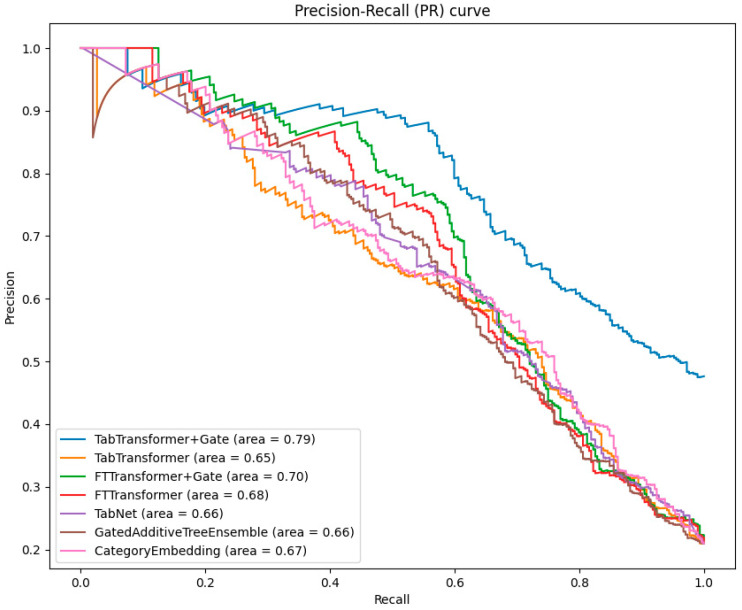
Comparative analysis of PR curves across models.

As can be seen from Figure 5, the TabTransformer+Gate model continues to perform best among all models, with an area under the PR curve of 0.79. This highlights the superiority of the gating mechanism in improving the precision of model predictions in the face of imbalanced datasets.

The area under the PR curve for the TabTransformer and FTTransformer is slightly lower than that of the TabTransformer+Gate and FTTransformer+Gate. However, this still indicates the effectiveness of the Transformer in handling imbalanced data.

**Figure 6:**
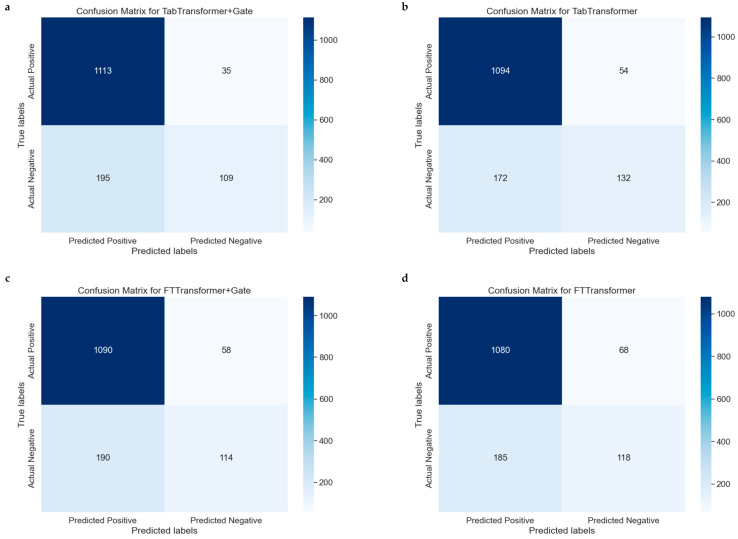
Comparative confusion matrices for multiple models. (**a**): Confusion matrix for the model combining the TabTransformer architecture with a gating mechanism. (**b**) Confusion matrix for the basic TabTransformer model. (**c**) Confusion matrix for the FTTransformer model enhanced with a gating mechanism. (**d**) Confusion matrix for the basic FTTransformer model.

The confusion matrix is a particularly helpful tool for evaluating the performance of classification models because it not only shows the number of samples correctly classified by the model but also reveals cases of misclassification. Figure 6a–d represent the confusion matrices for the TabTransformer, TabTransformer+Gate, FTTransformer, and FTTransformer+Gate models, respectively, indicating model performance.

The confusion matrices for the models are summarized as follows: The TabTransformer+Gate model (Figure 6a) correctly predicted 1094 true positives (TP) and 160 true negatives (TN). In comparison, the TabTransformer model (Figure 6b) predicted 1099 TPs and 140 TNs but misclassified 49 negatives as false positives (FP) and 164 positives as false negatives (FN). The FTTransformer+Gate model (Figure 6c) predicted 1090 TPs and 114 TNs, with 190 negatives misclassified as FPs and 58 positives as FNs. Lastly, the FTTransformer model (Figure 6d) predicted 1080 TPs and 118 TNs, misclassifying 185 negatives as FPs and 68 positives as FNs.

Compared to the TabTransformer, the TabTransformer+Gate model shows a slight improvement in correctly predicting positives and a noticeable increase in correctly predicting negatives, resulting in a more balanced performance. Similarly, the FTTransformer+Gate model improves in correctly predicting positives but slightly decreases in correctly predicting negatives, excelling in reducing false negatives at the cost of increased false positives. Overall, the inclusion of the gate mechanism enhances the models’ ability to correctly predict positives and reduce false negatives, though it slightly increases false positives. The choice of model with or without a gate depends on the specific need to balance positive and negative prediction accuracy, with the TabTransformer+Gate offering a more balanced performance and the FTTransformer+Gate prioritizing positive prediction accuracy.

**Figure 7:**
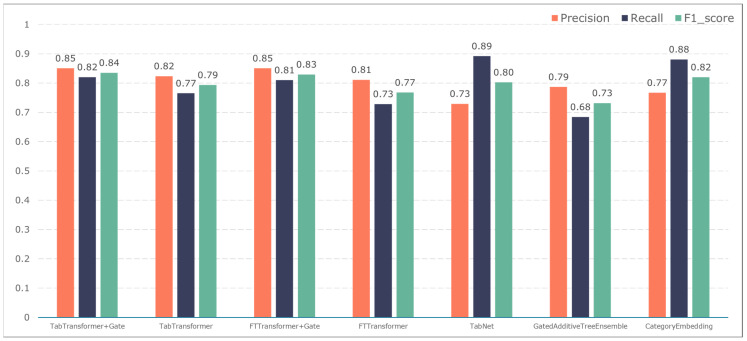
Model performance in predicting ischemic events.

**Figure 8:**
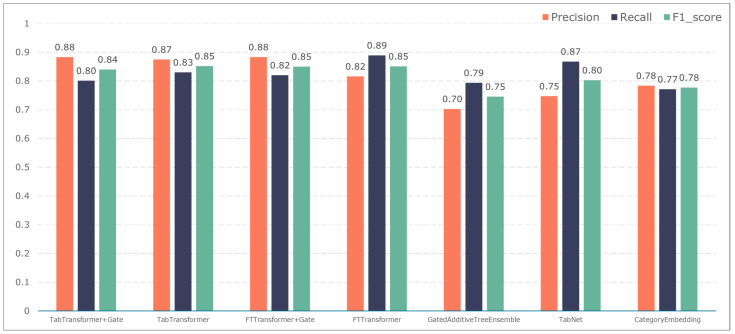
Model performance in predicting bleeding events.

**Figure 9:**
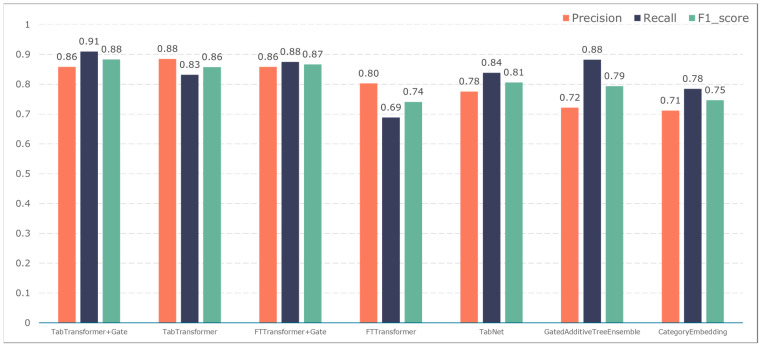
Model performance in predicting infection events.

**Figure 10:**
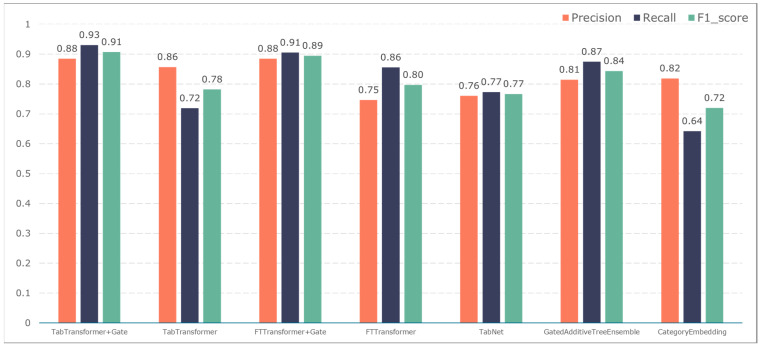
Model performance in predicting fever events.

**Figure 11:**
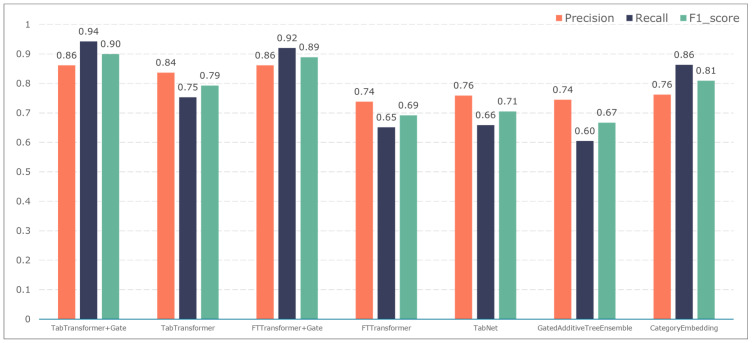
Model performance in predicting vascular reconstruction.

To further validate the gating performance, we also used F1-score, precision, and recall to evaluate the prediction performance of five adverse events: ischemic events, bleeding events, infectious events, febrile events, and revascularization. The results are shown in Figure 7, Figure 8, Figure 9, Figure 10 and Figure 11.

The F1-score constitutes the primary metric utilized within this study for assessing the performance of both entity recognition and entity relationship identification models. Essentially, it is calculated by combining precision and recall values. A high F1-score indicates exemplary model performance. These measurements can be computed as follows:

(9) $$Precision=\frac{TP}{TP+FP}$$

(10) $$Recall=\frac{TP\;}{TP+FN}$$

(11) $$F1_{score}=\frac{2*Precision*Recall}{Precision+Recal}$$

where *TP* is the number of positive samples that are correctly labelled as positive samples, *FP* is the number of negative samples that are incorrectly labelled as positive samples, and *FN* indicates the number of positive samples that are mistakenly labelled as negative samples.

The TabTransformer+Gate model maintains high precision, recall, and F1-scores across various prediction scenarios, demonstrating exceptional performance. This reflects the model’s effectiveness in feature learning and decision boundary delineation. Its advantage stems from the introduction of the Gate mechanism, which provides the model with more flexible representation capabilities when dealing with complex data structures. This flexibility helps the model exhibit better adaptability and robustness in predicting different types of adverse events.

Compared to the TabTransformer+Gate, the TabTransformer, while utilizing a similar foundational architecture, lacks the Gate mechanism, which may be why it performs slightly less well in certain scenarios. Other models, such as the FTTransformer+Gate, FTTransformer, TabNet, GatedAdditiveTreeEnsemble, and CategoryEmbedding, can achieve relatively satisfactory precision, recall, and F1-scores in prediction tasks. However, their overall performance reveals potential limitations in model complexity, feature representation, and generalization capabilities.

### 3.3. Implementation of the Design of Acute Coronary Syndrome Patient Management Platform

The Acute Coronary Syndrome Patient Management System, through the collection and analysis of real-time data, achieves the provision of timely feedback and intervention suggestions for patients, as well as offering real-time information on patient management for healthcare providers, thus realizing comprehensive management of patients with acute coronary syndrome. The system mainly consists of two modules: electronic health record entry and adverse event prediction.

**Figure 12:**
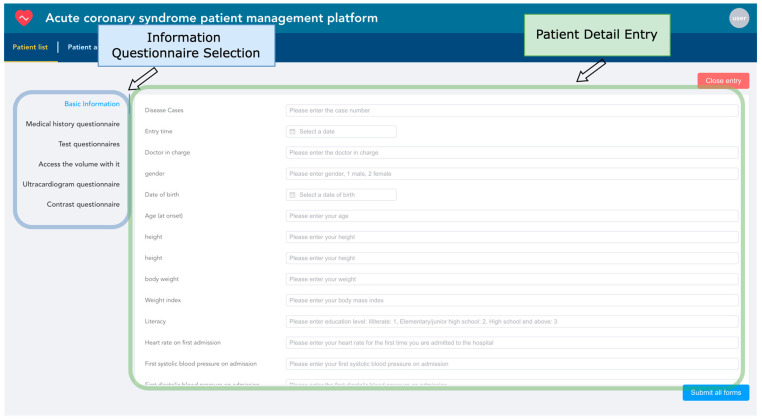
Patient information entry snapshot.

Electronic health records are entered based on the patient as the basic unit. The system uses the unique patient ID as the index condition, automatically associating the patient’s hospitalization information from multiple admissions. Each patient corresponds to one or several hospitalizations. As shown in Figure 12, information entry is conducted through six types of structured questionnaires. Within the blue box, healthcare providers can choose any category among basic information, medical history, laboratory test results, follow-up data, echocardiogram data, and angiography data to start entering patient information. If the patient ID already exists, the system will update with the newly entered data while retaining unchanged data to ensure the uniqueness of the information.

**Figure 13:**
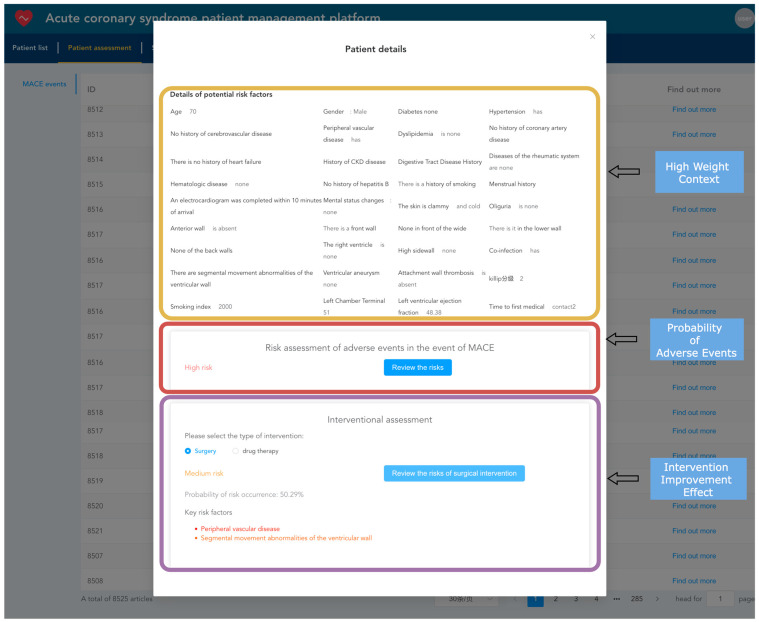
Patient risk prediction snapshot.

After importing electronic health records into the system, they can be viewed and analyzed. Healthcare providers can use the TabTransformer+Gated model, already loaded into the memory, to predict adverse events for patients, estimating the probability of short-term major adverse cardiovascular events occurring as well as the probability of such events occurring after interventions, such as surgical or medication treatments. As shown in Figure 13, the yellow box displays the context with the highest model weights during the prediction of adverse events. The red box shows the model’s predicted probability of the current patient experiencing an adverse event. The purple box shows the probability of an adverse event occurring after intervention treatments (such as surgical or medication interventions), highlighting the key risk factors that influence the outcome of intervention treatments.

## 4. Discussion

Significance of Research Results: Our study introduces an innovative approach to predicting MACEs in patients with ACS through the use of the TabTransformer model enhanced with gating mechanisms. Given the dynamic nature of ACS and its significant burden on global health, this study is crucial. Unlike traditional risk stratification models, our method provides a more detailed understanding of patient data. By incorporating gating mechanisms into the self-attention layers and feed-forward network layers, the TabTransformer+Gate model offers a more flexible and dynamic way to process and learn features from tabular data. It effectively learns and differentiates the importance of various features, which is particularly important for understanding the complex interactions and dependencies between features. This enhanced model architecture gives the TabTransformer+Gate superior performance and generalization capabilities when dealing with tabular data that contain complex relationships and diverse features.

Comparison with Previous Work: The study introduces the TabTransformer+Gate model to predict MACEs in patients with ACS. This model incorporates Transformer architecture enhanced with a gating mechanism, providing a significant improvement in processing and analyzing complex, high-dimensional datasets. This dynamic gating mechanism allows for robust predictions across varying datasets, showing superior generalization capabilities. It effectively captures complex interactions between features, similar to findings in other studies that utilized advanced machine learning models for medical data analysis [32]. Ultimately, the TabTransformer+Gate model offers more precise MACE risk assessments, aligning with enhancements in clinical decision-making and patient management strategies noted in comparative studies [33]. These advancements in AI applications in healthcare suggest a significant improvement over traditional risk assessment methods and other deep learning models.

By integrating the gating mechanism with the TabTransformer+Gate model, we not only improved the accuracy of MACE predictions but also provided a new perspective on the application of deep learning in the healthcare domain.

Clinical Significance: We developed a management platform specifically designed for ACS patients. Through the application of the TabTransformer+Gate model, medical professionals can more effectively identify patients at high risk, thereby providing timely and targeted intervention measures. This early identification and personalized treatment approach not only helps to reduce the risk of MACEs but also significantly improves treatment outcomes for patients while optimizing resource allocation within the healthcare system [34,35]. The platform can integrate various types of data, including real-time patient data, offering a more dynamic, responsive, and adaptable management approach for patient care. Through this platform, medical teams can monitor patients’ health status in real time and develop and adjust treatment plans based on model-predicted risk levels, ensuring that patients receive the most appropriate treatment and care. This integrated and systematic management approach not only improves treatment efficiency but also enhances patient satisfaction, providing a comprehensive and personalized treatment and management plan for ACS patients.

Limitations and Challenges: Although our TabTransformer+Gate model demonstrated excellent performance in predicting MACEs in patients with ACS, its effectiveness highly depends on the quality and comprehensiveness of the input data. This highlights the importance of accurate and thorough data collection in clinical settings [36]. Despite the model considering a wide range of variables, the continuous advancement in medical research means the model needs to be regularly updated and optimized to include the latest risk factors and clinical insights [37].

Future Directions: Future research will focus on enhancing the model’s predictive capability by integrating novel biomarkers and imaging data, which may further improve its accuracy and reliability in clinical applications [38]. Additionally, to ensure the model’s universality and effectiveness, a broader assessment of its applicability across different populations and healthcare settings is needed. Developing a user-friendly interface will be key to facilitating the adoption of this technology in daily clinical practice, allowing for healthcare providers to perform advanced risk predictions without needing to understand the model’s details [39]. Such an interface will also help promote communication and collaboration between interdisciplinary teams, further integrating the model into the clinical decision-making process.

Broader Impact: This study not only contributes to the field of digital health but also demonstrates the potential of machine learning technologies to bridge the gap between data science and clinical practice. By leveraging advanced data analysis techniques, our model offers new perspectives for predicting outcomes of ACS and other cardiovascular diseases, potentially transforming future disease management and treatment strategies. Moreover, the approach and principles of the model can be extended to other medical fields, providing a template for a wide range of disease predictions and health management [40]. With the development of technology and the increasing richness of medical data, we anticipate seeing further development.

## 5. Conclusions

This study successfully demonstrates the application of a Transformer model combined with a gating mechanism to predict MACEs in ACS patients. Integrating gating mechanisms into Transformer models provides a novel approach to understanding and analyzing structured tabular data in the medical field, providing insights beyond traditional approaches in terms of depth and accuracy. Depending on clinical priorities (e.g., maximizing true-positive identification versus ensuring high predictive accuracy), one can choose between gating and non-gating mechanisms. For situations where the lack of positive cases is critical, a gated model is preferable. For contexts that emphasize accurate positive predictions, ungated models may be more appropriate. Through rigorous data preprocessing, an innovative model design, and a comprehensive performance evaluation, our findings highlight some key achievements and implications for future research and clinical practice. Furthermore, developing a patient management platform that incorporates predictive models as a new means of treating ACS patients could significantly improve the personalization and precision of treatment. Such a platform can optimize patients’ treatment plans, enhance patient compliance and effectiveness, and simultaneously achieve the dynamic monitoring and management of health status, ultimately improving patients’ prognosis and quality of life.

## Figures and Tables

**Table 1:** Data distribution.

Data Types	Quantity	Proportion
Basic information	8	6%
Medical history	31	23.1%
Laboratory test results	30	22.3%
Follow-up	25	18.6%
ECHO	20	15%
Angiography	20	15%

**Table 2:** Model selection parameter setting.

Parameter	Value
Learning Rate	5 × 10^−6^
Input embedding dimension	128
Batch size	256
Epoch	200
Dropout	0.1
Attention heads	8
Attention blocks	6
Attention Dropout	0.1
AddNorm Dropout	0.1

## Data Availability

The data that support the findings of this study are available on request from the corresponding author. The data are not publicly available due to privacy or ethical restrictions.

## Appendix A

Appendix A provides a comprehensive overview of the feature variables utilized in this study. Each feature is meticulously documented with its name, type, and a brief description, ensuring clarity and transparency. This detailed catalog includes variables spanning six categories: basic information, medical history, laboratory test results, follow-up data, ECHO data, and angiography data. By offering this level of detail, Appendix A facilitates the reproducibility of the study and allows for other researchers to understand the variables’ significance and potential impact on the model’s performance. This thorough documentation supports further validation and application of our findings in various clinical and research settings.

**Table A1:** Detailed feature variable overview.

Variable Name	Category	Type	Description
Gender	Basic information	Categorical	The biological sex of the patient.
Birth Date	Basic information	Numerical	The date of birth of the patient.
Age at Onset	Basic information	Numerical	The age at which the patient first experienced symptoms.
Body Mass Index (BMI)	Basic information	Numerical	The body mass index of the patient.
Initial Heart Rate	Basic information	Numerical	The patient’s heart rate upon first admission.
Initial Systolic BP	Basic information	Numerical	The patient’s systolic blood pressure upon first admission.
Initial Diastolic BP	Basic information	Numerical	The patient’s diastolic blood pressure upon first admission.
Initial Oxygenation	Basic information	Numerical	The patient’s oxygenation level upon first admission.
History of Diabetes	Medical history	Categorical	Whether the patient has a history of diabetes.
History of Hypertension	Medical history	Categorical	Whether the patient has a history of hypertension.
History of Stroke	Medical history	Categorical	Whether the patient has a history of cerebrovascular disease.
History of PVD	Medical history	Categorical	Whether the patient has a history of peripheral vascular disease.
History of Dyslipidemia	Medical history	Categorical	Whether the patient has a history of dyslipidemia.
History of CAD	Medical history	Categorical	Whether the patient has a history of coronary artery disease.
History of CABG	Medical history	Categorical	Whether the patient has a history of coronary artery bypass graft surgery.
History of Heart Failure	Medical history	Categorical	Whether the patient has a history of heart failure.
History of COPD	Medical history	Categorical	Whether the patient has a history of chronic obstructive pulmonary disease.
History of CKD	Medical history	Categorical	Whether the patient has a history of chronic kidney disease.
History of GI Ulcer	Medical history	Categorical	Whether the patient has a history of gastrointestinal ulcers.
Rheumatic Diseases	Medical history	Categorical	Whether the patient has rheumatic diseases.
Hematologic Diseases	Medical history	Categorical	Whether the patient has hematologic diseases.
History of Hepatitis B	Medical history	Categorical	Whether the patient has a history of hepatitis B.
Smoking History	Medical history	Categorical	Whether the patient has a history of smoking.
Smoking Index	Medical history	Numerical	The smoking index of the patient.
Menstrual History	Medical history	Categorical	The patient’s menstrual history.
Age at Menopause	Medical history	Numerical	The age at which the patient experienced menopause.
Alcohol Consumption History	Medical history	Categorical	Whether the patient has a history of alcohol consumption.
History of Chest Pain	Medical history	Categorical	Whether the patient has a history of chest pain.
Angina Episodes > 2 in 24 h Before Admission	Medical history	Categorical	Whether the patient experienced more than two episodes of angina in the 24 h before admission.
Time to First Medical Contact	Medical history	Numerical	The time from symptom onset to first medical contact.
Symptoms at Onset	Medical history	Categorical	The symptoms experienced by the patient at onset.
Chest Pain	Medical history	Categorical	Whether the patient experienced chest pain.
Radiating Pain	Medical history	Categorical	Whether the patient experienced radiating pain.
Nausea and Vomiting	Medical history	Categorical	Whether the patient experienced nausea and vomiting.
Profuse Sweating	Medical history	Categorical	Whether the patient experienced profuse sweating.
Shortness of Breath	Medical history	Categorical	Whether the patient experienced shortness of breath.
Loss of Consciousness	Medical history	Categorical	Whether the patient experienced loss of consciousness.
Duration of Symptoms	Medical history	Numerical	The duration of the symptoms experienced by the patient.
ECG Completed within 10 Minutes of Arrival	Medical history	Categorical	Whether an ECG was completed within 10 min of the patient’s arrival.
High-Sensitivity CRP	Laboratory test	Numerical	The level of high-sensitivity C-reactive protein in the patient.
BNP	Laboratory test	Numerical	The level of brain natriuretic peptide in the patient.
cTnI	Laboratory test	Numerical	The level of cardiac troponin I in the patient.
PCT	Laboratory test	Numerical	The level of procalcitonin in the patient.
IL-6	Laboratory test	Numerical	The level of interleukin-6 in the patient.
D-Dimer	Laboratory test	Numerical	The level of D-dimer in the patient.
pH	Laboratory test	Numerical	The pH level of the patient’s blood.
Albumin	Laboratory test	Numerical	The level of albumin in the patient.
ALT	Laboratory test	Numerical	The level of alanine aminotransferase in the patient.
WBC Count	Laboratory test	Numerical	The white blood cell count of the patient.
RDW	Laboratory test	Numerical	The red cell distribution width of the patient.
RBC Count	Laboratory test	Numerical	The red blood cell count of the patient.
Hematocrit (HCT)	Laboratory test	Numerical	The hematocrit level of the patient.
Creatinine (Cr)	Laboratory test	Numerical	The creatinine level of the patient.
Creatine Kinase (CK)	Laboratory test	Numerical	The creatine kinase level of the patient.
Potassium (K)	Laboratory test	Numerical	The potassium level of the patient.
Calcium (Ca)	Laboratory test	Numerical	The calcium level of the patient.
Total Cholesterol (TC)	Laboratory test	Numerical	The total cholesterol level of the patient.
LDL-C	Laboratory test	Numerical	The low-density lipoprotein cholesterol level of the patient.
HDL-C	Laboratory test	Numerical	The high-density lipoprotein cholesterol level of the patient.
Triglycerides (TG)	Laboratory test	Numerical	The triglyceride level of the patient.
Glucose	Laboratory test	Numerical	The glucose level of the patient.
Fibrinogen (FIB)	Laboratory test	Numerical	The fibrinogen level of the patient.
Hemoglobin (Hb)	Laboratory test	Numerical	The hemoglobin level of the patient.
Platelet Count (PLT)	Laboratory test	Numerical	The platelet count of the patient.
Blood Urea Nitrogen (BUN)	Laboratory test	Numerical	The blood urea nitrogen level of the patient.
Uric Acid (UA)	Laboratory test	Numerical	The uric acid level of the patient.
Triiodothyronine (T3)	Laboratory test	Numerical	The triiodothyronine level of the patient.
Thyroxine (T4)	Laboratory test	Numerical	The thyroxine level of the patient.
Free Thyroxine (FT4)	Laboratory test	Numerical	The free thyroxine level of the patient.
Quality of Life	Follow-up	Categorical	The patient’s perceived quality of life.
Regular Follow-up	Follow-up	Categorical	Whether the patient has regular follow-ups.
Strict Smoking Cessation	Follow-up	Categorical	Whether the patient strictly abstains from smoking.
Blood Pressure Control	Follow-up	Categorical	Whether the patient’s blood pressure is controlled.
Blood Glucose Control	Follow-up	Categorical	Whether the patient’s blood glucose is controlled.
Deceased	Follow-up	Categorical	Whether the patient is deceased.
Cause of Death	Follow-up	Categorical	The cause of the patient’s death.
Recurrent MI	Follow-up	Categorical	Whether the patient has had a recurrent myocardial infarction.
Number of Recurrent MIs	Follow-up	Numerical	The number of recurrent myocardial infarctions the patient has had.
Arrhythmia	Follow-up	Categorical	Whether the patient has experienced arrhythmia.
Time of Arrhythmia Onset	Follow-up	Numerical	The time when the patient experienced arrhythmia.
Atrial Fibrillation	Follow-up	Categorical	Whether the patient has atrial fibrillation.
AV Block	Follow-up	Categorical	Whether the patient has atrioventricular block.
Bundle Branch Block	Follow-up	Categorical	Whether the patient has bundle branch block.
Ventricular Arrhythmia	Follow-up	Categorical	Whether the patient has ventricular arrhythmia.
Arrhythmia Treatment	Follow-up	Categorical	The treatment for the patient’s arrhythmia.
Heart Failure	Follow-up	Categorical	Whether the patient has heart failure.
Time of Heart Failure Onset	Follow-up	Numerical	The time when the patient experienced heart failure.
Stroke	Follow-up	Categorical	Whether the patient has experienced a stroke.
Time of Stroke Onset	Follow-up	Numerical	The time when the patient experienced a stroke.
Type of Stroke	Follow-up	Categorical	The type of stroke the patient experienced.
Bleeding	Follow-up	Categorical	Whether the patient has experienced bleeding.
Time of Bleeding Onset	Follow-up	Numerical	The time when the patient experienced bleeding.
Bleeding Classification	Follow-up	Categorical	The classification of the patient’s bleeding.
Current Oral Medications	Follow-up	Categorical	The current oral medications the patient is taking.
Ascending Aorta Diameter	ECHO	Numerical	The diameter of the patient’s ascending aorta.
Aortic Root Diameter	ECHO	Numerical	The diameter of the patient’s aortic root.
Right Ventricular Outflow Tract	ECHO	Numerical	The measurement of the right ventricular outflow tract.
Right Ventricular Anteroposterior Diameter	ECHO	Numerical	The anteroposterior diameter of the right ventricle.
Aortic Diameter	ECHO	Numerical	The diameter of the patient’s aorta.
Left Ventricular End-Diastolic Diameter	ECHO	Numerical	The end-diastolic diameter of the left ventricle.
Left Ventricular End-Systolic Diameter	ECHO	Numerical	The end-systolic diameter of the left ventricle.
Left Atrial Anteroposterior Diameter	ECHO	Numerical	The anteroposterior diameter of the left atrium.
Interventricular Septal End-Diastolic Thickness	ECHO	Numerical	The end-diastolic thickness of the interventricular septum.
Right Atrial Vertical Diameter	ECHO	Numerical	The vertical diameter of the right atrium.
Right Atrial Horizontal Diameter	ECHO	Numerical	The horizontal diameter of the right atrium.
Left Ventricular Ejection Fraction (LVEF)	ECHO	Numerical	The ejection fraction of the left ventricle.
Left Ventricular Shortening Fraction	ECHO	Numerical	The shortening fraction of the left ventricle.
Aortic Valve Regurgitation	ECHO	Categorical	Whether the patient has aortic valve regurgitation.
Aortic Valve Calcification	ECHO	Categorical	Whether the patient has aortic valve calcification.
Ventricular Wall Thinning	ECHO	Categorical	Whether the patient has thinning of the ventricular wall.
Location of Wall Thinning: Anterior Septum	ECHO	Categorical	Whether the wall thinning is located at the anterior septum.
Location of Wall Thinning: Septum	ECHO	Categorical	Whether the wall thinning is located at the septum.
Location of Wall Thinning: Anterior Wall	ECHO	Categorical	Whether the wall thinning is located at the anterior wall.
Location of Wall Thinning: Lateral Wall	ECHO	Categorical	Whether the wall thinning is located at the lateral wall.
Admission Heart Rate (HR1)	Angiography	Numerical	The patient’s heart rate upon admission.
Admission Systolic BP (SP1)	Angiography	Numerical	The patient’s systolic blood pressure upon admission.
Admission Diastolic BP (DP1)	Angiography	Numerical	The patient’s diastolic blood pressure upon admission.
Initial Oxygen Saturation (Spo21)	Angiography	Numerical	The patient’s oxygen saturation level upon first measurement.
Use of Vasopressors During Surgery	Angiography	Categorical	Whether vasopressors were used during surgery.
Intraoperative Complications	Angiography	Categorical	The complications that occurred during surgery.
Surgical Procedure	Angiography	Categorical	Whether the patient underwent surgery.
Intraoperative Contrast Agent Dosage	Angiography	Numerical	The dosage of contrast agent used during surgery.
Intraoperative Heparin Dosage	Angiography	Numerical	The dosage of heparin used during surgery.
Coronary Dominance	Angiography	Categorical	The dominance pattern of the coronary arteries.
Culprit Vessel Characteristics	Angiography	Categorical	The characteristics of the culprit vessel.
TIMI Flow Grade Pre- and Post-Intervention in Culprit Vessel	Angiography	Categorical	The TIMI flow grade in the culprit vessel before and after intervention.
Coronary Lesion Location and Stenosis Degree	Angiography	Categorical	The location and degree of stenosis of the coronary lesion.
Number and Type of Stents Implanted	Angiography	Numerical	The number and types of stents implanted.
Use of Thrombus Aspiration During Surgery	Angiography	Categorical	Whether thrombus aspiration was used during surgery.
Planned Second PCI	Angiography	Categorical	Whether a second PCI is planned.
Medication Adherence at Discharge	Angiography	Categorical	The adherence to prescribed medications at discharge.
Use of Temporary Pacemaker, ECMO, IABP During Surgery	Angiography	Categorical	Whether a temporary pacemaker, ECMO, or IABP was used during surgery.
Management of Intraoperative Complications	Angiography	Categorical	The management strategies for complications during surgery.
Use of Intravascular Imaging During Stent Placement	Angiography	Categorical	Whether intravascular imaging was used during stent placement.
